# Supplementary material for: Seroprevalence and associated risk factors of brucellosis, Rift Valley fever and Q fever among settled and mobile agro-pastoralist communities and their livestock in Chad
Source: PLoS Negl Trop Dis. 2023 Jun 23;17(6):e0011395. doi: 10.1371/journal.pntd.0011395 (PMC10351688; doi:10.1371/journal.pntd.0011395)
Supplement: S1 Table — NA stands for a missing observation. (DOCX) [file pntd.0011395.s001.docx]

**S1 Table.** Number and proportion of animals sampled from each age category, separated by species. NA stands for a missing observation.

| **Species** | **Age category** | **Count** | **Proportion %** |
| --- | --- | --- | --- |
| Bovine | < 3 | 206 | 53.1 |
|  | ≥ 3 | 182 | 46.9 |
| Sheep | < 3 | 203 | 55.0 |
|  | ≥ 3 | 165 | 44.7 |
|  | NA | 1 | 0.3 |
| Goat | < 3 | 75 | 48.4 |
|  | ≥ 3 | 79 | 51.0 |
|  | NA | 1 | 0.6 |
| Horse | < 3 | 3 | 3.7 |
|  | ≥ 3 | 79 | 96.3 |
| Donkey | < 3 | 8 | 17 |
|  | ≥ 3 | 39 | 83.0 |
