# Supplementary material for: Seroprevalence and associated risk factors of brucellosis, Rift Valley fever and Q fever among settled and mobile agro-pastoralist communities and their livestock in Chad
Source: PLoS Negl Trop Dis. 2023 Jun 23;17(6):e0011395. doi: 10.1371/journal.pntd.0011395 (PMC10351688; doi:10.1371/journal.pntd.0011395)
Supplement: S2 Table — NA stands for a missing observation. (DOCX) [file pntd.0011395.s002.docx]

| **Species** | **Sex** | **Count** | **Proportion %** |
| --- | --- | --- | --- |
| Bovine | Male | 136 | 35.1 |
|  | Female | 250 | 64.4 |
|  | NA | 2 | 0.5 |
| Sheep | Male | 98 | 26.6 |
|  | Female | 269 | 72.9 |
|  | NA | 2 | 0.5 |
| Goat | Male | 35 | 22.6 |
|  | Female | 118 | 76.1 |
|  | NA | 2 | 1.3 |
| Horse | Male | 61 | 74.4 |
|  | Female | 21 | 25.6 |
| Donkey | Male | 31 | 66.0 |
|  | Female | 16 | 34.0 |
| NA | Female | 2 | 100 |

**S2 Table**. Number (count) and proportion of animals sampled by species and sex. NA stands for a missing observation.
