# Supplementary material for: Seroprevalence and associated risk factors of brucellosis, Rift Valley fever and Q fever among settled and mobile agro-pastoralist communities and their livestock in Chad
Source: PLoS Negl Trop Dis. 2023 Jun 23;17(6):e0011395. doi: 10.1371/journal.pntd.0011395 (PMC10351688; doi:10.1371/journal.pntd.0011395)
Supplement: S3 Table — Number of samples tested (Tested), number of seropositive samples (Positive), apparent seroprevalence (AP), and true seroprevalence using Bayesian modeling adjusting for imperfect test characteristics and clustering (TP). Data presented for the regions Danamadji and Yao, as well as combined for both regions. (DOCX) [file pntd.0011395.s003.docx]

**S3 Table. Human and animal disease seroprevalence (%).** Number of samples tested (Tested), number of seropositive samples (Positive), apparent seroprevalence (AP), and true seroprevalence using Bayesian modeling adjusting for imperfect test characteristics and clustering (TP). Data presented for the regions Danamadji and Yao, as well as combined for both regions.

|  | | **Combined** | | | | **Danamadji** | | | | **Yao** | | | |
| --- | --- | --- | --- | --- | --- | --- | --- | --- | --- | --- | --- | --- | --- |
| **Disease** | **Species** | **Tested** | **Positive** | **AP % (95%CI^#^)** | **TP  (95%CrI*)** | **Tested** | **Positive** | **AP % (95%CI^#^)** | **TP % (95%CrI*)** | **Tested** | **Positive** | **AP % (95%CI^#^)** | **TP % (95%CrI*)** |
| **Brucellosis** | Human | 959 | 18 | 1.9 (1.2-2.9) | 0.2 (0-1.1) | 569 | 5 | 0.9 (0.4-2.0) | 0.2 (0-1.2) | 390 | 13 | 3.3 (2.0-5.6) | 0.5 (0-3.1) |
|  | All animal species | 1041 | 59 | 5.7 (4.4-7.2) | 0.3 (0-1.5) | 558 | 20 | 3.6 (2.3-5.5) | 0.5 (0-2.9) | 483 | 39 | 8.1 (6.0-10.8) | 0.8 (0-4.1) |
|  | Cattle | 387 | 15 | 3.9 (2.4-6.3) | 0.5 (0-2.3) | 300 | 9 | 3.0 (1.6-5.6) | 0.3 (0-2.7) | 87 | 6 | 6.9 (3.2-14.2) | 2.3 (0-11.5) |
|  | Sheep | 369 | 27 | 7.3 (5.1-10.4) | 0.8 (0-3.8) | 240 | 11 | 4.6 (2.6-8.0) | 0.8 (0-4.6) | 129 | 16 | 12.4 (7.8-19.2) | 4.7 (0-14.0) |
|  | Goat | 155 | 13 | 8.4 (5.0-13.8) | 1.9 (0-9.0) | 12 | 0 | **-** | **-** | 143 | 13 | 9.1 (5.4-14.9) | 2.8 (0-10.5) |
|  | Equine | 128 | 4 | 3.1 (1.2-7.8) | 0.8 (0-3.9) | 6 | 0 | **-** | **-** | 122 | 4 | 3.3 (1.3-8.1) | 0 (0-4.1) |
| **Q fever** | Human | 960 | 381 | 39.7 (36.6-42.8) | 49.1 (38.9-58.8) | 570 | 269 | 47.2 (43.1-51.3) | 63.0 (52.3-74.7) | 390 | 112 | 28.7 (24.5-33.4) | 35.1 (22.3-46.7) |
|  | All animal species | 975 | 131 | 13.4 (11.4-15.7) | 12.8 (9.7-16.4) | 498 | 58 | 11.6 (9.1-14.8) | 11.4 (7.6-15.9) | 477 | 73 | 15.3 (12.4-18.8) | 14.5 (10.1-19.7) |
|  | Cattle | 353 | 32 | 9.1 (6.5-12.5) | 7.1 (3.4-11.6) | 267 | 21 | 7.9 (5.2-11.7) | 5.6 (2.2-10.5) | 86 | 11 | 12.8 (7.3-21.5) | 12.8 (4.7-25.6) |
|  | Sheep | 341 | 63 | 18.5 (14.7-22.9) | 19.1 (13.2-25.2) | 213 | 36 | 16.9 (12.5-22.5) | 16.9 (10.3-24.9) | 128 | 27 | 21.1 (14.9-29.0) | 21.1 (12.5-32.8) |
|  | Goat | 152 | 29 | 19.1 (13.6-26.1) | 17.1 (8.6-25.7) | 11 | 1 | - | - | 141 | 28 | 19.9 (14.1-27.2) | 17.7 (9.2-27.7) |
|  | Equine | 127 | 7 | 5.5 (2.7-10.9) | 3.1 (0-9.4) | 7 | 0 | - | - | 120 | 7 | 5.8 (2.9-11.6) | 4.2 (0-11.7) |
| **Rift Valley fever** | Human | 954 | 280 | 29.4 (26.5-32.3) | 28.1 (23.4-33.3) | 559 | 149 | 26.7 (23.2-30.5) | 25.9 (19.9-31.7) | 395 | 131 | 33.2 (28.7-38.0) | 32.9 (25.1-39.7) |
|  | All animal species | 1002 | 128 | 12.8 (10.8-15.0) | 10.2 (7.6-13.4) | 523 | 85 | 16.3 (13.3-19.7) | 13.6 (9.6-18.2) | 479 | 43 | 9.0 (6.7-11.9) | 7.9 (4.8-11.5) |
|  | Cattle | 370 | 49 | 13.2 (10.2-17.1) | 9.5 (5.7-14.3) | 283 | 36 | 12.7 (9.3-17.1) | 9.2 (5.3-14.1) | 87 | 13 | 14.9 (8.9-23.9) | 11.5 (3.4-23.0) |
|  | Sheep | 349 | 60 | 17.2 (13.6-21.5) | 15.5 (10.6-21.5) | 221 | 45 | 20.4 (15.6-26.2) | 18.6 (11.8-26.2) | 128 | 15 | 11.7 (7.2-18.4) | 11.7 (4.7-21.1) |
|  | Goat | 152 | 6 | 3.9 (1.8-8.3) | 3.9 (0.7-9.2) | 12 | 2 | - | - | 140 | 4 | 2.9 (1.1-7.1) | 2.9 (0-7.9) |
|  | Equine | 129 | 12 | 9.3 (5.4-15.6) | 7.8 (2.3-15.5) | 7 | 2 | - | - | 122 | 10 | 8.2 (4.5-14.4) | 6.6 (1.6-15.6) |

^#^ 95% Confidence interval

*95% Credibility interval
