# Supplementary material for: Seroprevalence and associated risk factors of brucellosis, Rift Valley fever and Q fever among settled and mobile agro-pastoralist communities and their livestock in Chad
Source: PLoS Negl Trop Dis. 2023 Jun 23;17(6):e0011395. doi: 10.1371/journal.pntd.0011395 (PMC10351688; doi:10.1371/journal.pntd.0011395)
Supplement: S4 Table — (DOCX) [file pntd.0011395.s004.docx]

**S4 Table:** Univariable analysis results of risk factors tested for human brucellosis seropositivity in Yao and Danamadji, Chad.

| Variable | Odds ratio (95% CI), p-value |
| --- | --- |
| Animal brucellosis apparent prevalence | 18.0 (2e-04;2e+06), 0.629 |
| Age as count | 1.0 (1;1), 0.765 |
| Camp [ref] vs village | 1.0 (0.3;3.3), 0.936 |
| Male [ref] vs female | 0.3 (0.06;1.48), 0.135 |
| Q-fever co-infection present | 1.0 (0.5;3.5), 0.659 |
| RVF co-infection present | 0.6 (0.2;2.0), 0.413 |
