# Supplementary material for: Seroprevalence and associated risk factors of brucellosis, Rift Valley fever and Q fever among settled and mobile agro-pastoralist communities and their livestock in Chad
Source: PLoS Negl Trop Dis. 2023 Jun 23;17(6):e0011395. doi: 10.1371/journal.pntd.0011395 (PMC10351688; doi:10.1371/journal.pntd.0011395)
Supplement: S5 Table — (DOCX) [file pntd.0011395.s005.docx]

**S5 Table:** Univariable analysis results risk factors tested for animal brucellosis seropositivity in Yao and Danamadji, Chad.

|  | |
| --- | --- |
| Variable | **Odds ratio (95% CI), p-value** |
| Human brucellosis apparent prevalence | 7.8 (3e-02;2e+03 ), 0.470 |
| Species [ref=bovine]: |  |
| Equine | 0.5 (0.2;1.7), 0.257 |
| Small ruminants | 1.6 (0.8;3.1), 0.186 |
| Rvf co-infection present | Model did not converge |
| Q-fever co-infection present | 1.6 (0.8;3.3), 0.165 |
| Camp [ref] vs village | 0.7 (0.4;1.3), 0.267 |
| Sex: male [ref] vs female | 2.4 (1.2;4.7), 0.00938 |
| Age: <3 [ref] vs 3 and above | - 1. (0.6;2.0), 0.707 |
