# Supplementary material for: Seroprevalence and associated risk factors of brucellosis, Rift Valley fever and Q fever among settled and mobile agro-pastoralist communities and their livestock in Chad
Source: PLoS Negl Trop Dis. 2023 Jun 23;17(6):e0011395. doi: 10.1371/journal.pntd.0011395 (PMC10351688; doi:10.1371/journal.pntd.0011395)
Supplement: S6 Table — (DOCX) [file pntd.0011395.s006.docx]

**S6 Table:** Univariable analysis results risk factors tested for human Q fever seropositivity in Yao and Danamadji, Chad.

| Variable | Odds ratio (95% CI), p value |
| --- | --- |
| Animal Q fever apparent prevalence | 1.3 (0.27;6.49), 0.737 |
| Age as count | 0.99 (0.98;1.00), 0.0178 |
| Camp [ref] vs village | 1.1 (0.82;1.41), 0.611 |
| Male [ref] vs female | 1.2 (0.84;1.69), 0.319 |
| RVF co-infection present | 1.0 (0.75;1.43), 0.836 |
| Brucellosis co-infection present | 1.6 (0.56;4.48), 0.387 |
