# Supplementary material for: Seroprevalence and associated risk factors of brucellosis, Rift Valley fever and Q fever among settled and mobile agro-pastoralist communities and their livestock in Chad
Source: PLoS Negl Trop Dis. 2023 Jun 23;17(6):e0011395. doi: 10.1371/journal.pntd.0011395 (PMC10351688; doi:10.1371/journal.pntd.0011395)
Supplement: S7 Table — (DOCX) [file pntd.0011395.s007.docx]

**S7 Table:** Univariable analysis results is factors tested for animal Q fever seropositivity in Yao and Danamadji, Chad.

|  | |
| --- | --- |
| Variable | **Odds ratio (95% CI), p value** |
| Human Q fever apparent prevalence | 0.9 (0.3;2.6), 0.843 |
| Species [ref=Bovine]: |  |
| Equine | 0.6 (0.2;1.4), 0.209 |
| Small ruminants | 2.2 (1.4;3.6), 0.000549 |
| RVF co-infection present | 1.3 (0.8;2.3), 0.276 |
| Brucellosis co-infection present | 1.7 (0.9;3.4), 0.125 |
| Camp [ref] vs Village | 0.9 (0.6;1.4), 0.735 |
| Sex: male [ref] vs female | 1.8 (1.2;2.8), 0.00641 |
| Age: <3 [ref] vs 3 and above | 1.0 (0.7;1.5), 0.834 |
