# Supplementary material for: Seroprevalence and associated risk factors of brucellosis, Rift Valley fever and Q fever among settled and mobile agro-pastoralist communities and their livestock in Chad
Source: PLoS Negl Trop Dis. 2023 Jun 23;17(6):e0011395. doi: 10.1371/journal.pntd.0011395 (PMC10351688; doi:10.1371/journal.pntd.0011395)
Supplement: S8 Table — (DOCX) [file pntd.0011395.s008.docx]

**S8 Table:** Univariable analysis results risk factors tested for human RVF seropositivity in Yao and Danamadji, Chad.

| Variables | Odds ratio (95% CI), p value |
| --- | --- |
| Animal RVF apparent prevalence | 4.0 (1.3;12.3), 0.0137 |
| Age as count | 1.02 (1.01;1.03), 2.64e-05 |
| Camp [ref] vs village | 1.1 (0.8;1.5), 0.401 |
| Male [ref] vs female | 0.7 (0.5;1.0), 0.0507 |
| Q-fever co-infection present | 1.0 (0.7;1.3), 0.826 |
| Brucellosis co-infection present | 0.6 (0.2;2.0), 0.428 |
