# Supplementary material for: Seroprevalence and associated risk factors of brucellosis, Rift Valley fever and Q fever among settled and mobile agro-pastoralist communities and their livestock in Chad
Source: PLoS Negl Trop Dis. 2023 Jun 23;17(6):e0011395. doi: 10.1371/journal.pntd.0011395 (PMC10351688; doi:10.1371/journal.pntd.0011395)
Supplement: S9 Table — (DOCX) [file pntd.0011395.s009.docx]

**S9 Table:** Risk factors tested for animal RVF seropositivity in Yao and Danamadji, Chad.

| Animal rift valley fever | |
| --- | --- |
| Variables | **Odds ratio (95% CI), p value** |
| Human RVF apparent prevalence | 12.0 (2.9;52.7), 0.00069 |
| Species [ref=bovine]: |  |
| Equine | 0.9 (0.4;2.1), 0.841 |
| Small ruminants | 1.1 (0.6;1.8), 0.849 |
| Camp [ref] vs village | 1.1 (0.7;1.6), 0.667 |
| Sex: male [ref] vs female | 1.0 (0.7;1.6), 0.885 |
| Age: <3 [ref] vs 3 and above | 1.4 (0.9;2.2), 0.101 |
| Q-fever co-infection present | 1.4 (0.8;2.4), 0.231 |
| Brucellosis co-infection present | 0.1 (0.0;1.0), 0.0443 |
