# Supplementary figures and images for: Seroprevalence and associated risk factors of brucellosis, Rift Valley fever and Q fever among settled and mobile agro-pastoralist communities and their livestock in Chad
Source: PLoS Negl Trop Dis. 2023 Jun 23;17(6):e0011395. doi: 10.1371/journal.pntd.0011395 (PMC10351688; doi:10.1371/journal.pntd.0011395)

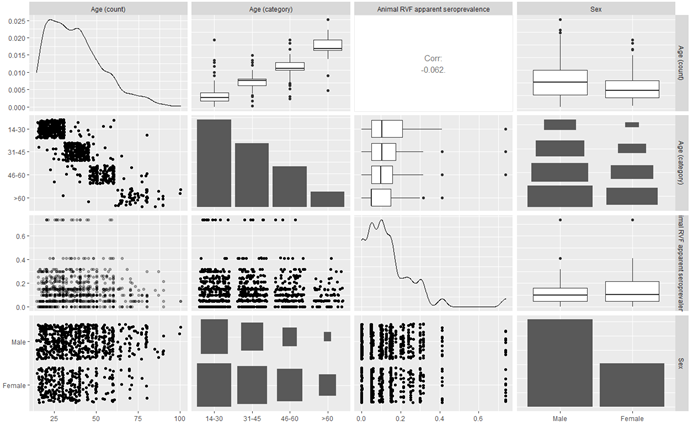

Supplement: S1 Fig — These variables were then implemented in the respective multivariable models. No strong correlation was found for any combination, except for Age (count) and Age (category), which were never used in the same models. The age is presented in years and the animal RVF apparent seroprevalence in %. (TIF) [file pntd.0011395.s010.tif]

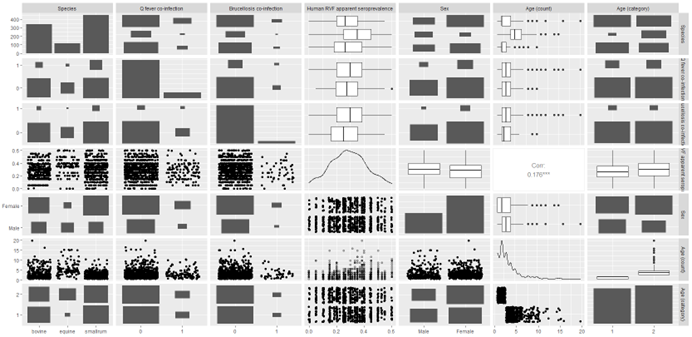

Supplement: S2 Fig — These variables were then implemented in the respective multivariable models. No strong correlation was found for any combination, except for Age (count) and Age (category), which were never used in the same models. The age is presented in years and the human RVF apparent seroprevalence in %. (PNG) [file pntd.0011395.s011.png]
