# Supplementary material for: Seroprevalence and associated risk factors of brucellosis, Rift Valley fever and Q fever among settled and mobile agro-pastoralist communities and their livestock in Chad
Source: PLoS Negl Trop Dis. 2023 Jun 23;17(6):e0011395. doi: 10.1371/journal.pntd.0011395 (PMC10351688; doi:10.1371/journal.pntd.0011395)
Supplement: S1 Methods — (DOCX) [file pntd.0011395.s012.docx]

S1 METHODOLOGY FOR THE MANUSCRIPT

*Seroprevalence and associated risk factors of brucellosis, Rift Valley fever and Q fever among settled and mobile agro-pastoralists communities and their livestock in Chad*

#### Estimation of true seroprevalence using a Bayesian framework and accounting for clustering

We first calculated the apparent seroprevalence as the number of individuals tested positive by the corresponding diagnostic tests divided by the total number of individuals tested. Confidence intervals around the prevalence estimates were calculated using the Clopper-Pearson method (92).

For the calculation of the true seroprevalence for each cluster (village or camp), we infer the sensitivity (Se) and specificity (Sp) of the different diagnostic tests for humans and animals from literature (Supplementary table X). The relation between the apparent seroprevalence (ap) and true seroprevalence (p) is described by:

$\boldsymbol{ap}_{\boldsymbol{j}}\boldsymbol{=}\boldsymbol{p}_{\boldsymbol{j}}\boldsymbol{\times}\boldsymbol{Se}\boldsymbol{+ (}\boldsymbol{1}\boldsymbol{-}\boldsymbol{p}_{\boldsymbol{j}}\boldsymbol{)\times(}\boldsymbol{1}\boldsymbol{+}\boldsymbol{Sp}\boldsymbol{)}$ ***Eq. 1***

where apparent seroprevalence (ap) in group *j* is the result of the true seroprevalence (p), the sensitivity (Se) and specificity (Sp) of the diagnostic tests. Hence, the true seroprevalence is calculated as follows, applying the Rogan-Gladen estimator (93):

| $\boldsymbol{p}_{\boldsymbol{j}}\boldsymbol{= (}\boldsymbol{ap}_{\boldsymbol{j}}\boldsymbol{+Sp-1)/(Se-1+Sp)}$ | ***Eq. 2*** |
| --- | --- |

Further, as the method for correcting the apparent seroprevalence towards a true seroprevalence by Rogan and Gladen has it's limitation when the apparent prevalence is rather small, we use a Bayesian approach for calculating the true seroprevalence in the absence of perfect diagnostic tests in line with recommendations of the HOTLINE (Harmonisation Of Transmissible disease Interpretation in the EU) project and as compared by other studies before (63,71). We infer the sensitivity and specificity of the different tests from literature (Supplementary table 1). We assume that the sensitivity and specificity were following beta distribution where the parameters (Se1, Se2 Sp1, Sp2) based on the observed values from literature:

| $\boldsymbol{Se \sim beta(Se}\boldsymbol{1, Se}\boldsymbol{2)}$  $\boldsymbol{Sp \sim beta(Sp}\boldsymbol{1, Sp}\boldsymbol{2)}$ | *Eq. 3* |
| --- | --- |

Similarly, the proportion of positive individuals (y) in a group of n individuals (cluster, region) followed a binomial distribution:

| $\boldsymbol{y \sim Binomial(n, ap)}$ | *Eq. 4* |
| --- | --- |

The apparent prevalence follows Equation 1, with a non-informative parameter of *beta(1,1)* for the true prevalence (p). Analyses are performed using the Rstan (94). Sampling is performed with a "NUTS" sampler (No-U-Turn sampler) (95). The true prevalence was calculated as *y* over *n*. Example model code is provided in supporting information R script 1 and 2 (PDFs).

The cluster sampling design delivers hierarchically structured data, where individual animals or humans are considered level 1 units, and the cluster level 2 units. To account for clustering design and considering dependence of individuals (i) within the clusters (j) we use logistic random-effect models (Eq. 5) implemented in the lme4 package (96,97).

| $\boldsymbol{logit(}\boldsymbol{Y}_{\boldsymbol{ij}}\boldsymbol{) =}\boldsymbol{X}_{\boldsymbol{ij}}\boldsymbol{\beta}_{\boldsymbol{0}}\boldsymbol{+}\boldsymbol{Z}_{\boldsymbol{ij}}\boldsymbol{b}_{\boldsymbol{j}}\boldsymbol{+}\boldsymbol{e}_{\boldsymbol{ij}}$ | *Eq. 5* |
| --- | --- |
|  |  |

Where individuals (level 1, i) are part of clusters (level 2, j). Random effect b varies across clusters (j). We reported the inverse logit of the intercept of the model ($\beta_{0}$) and its confidence interval as adjusted seroprevalence. We calculated the true seroprevalence for the entire dataset and stratified per region (Yao and Danamadji).

**Supplementary table X.** Human and animal test sensitivity and specificity from peer-reviewed and published literature. For animal test sensitivity and specificity, evaluation of cattle samples were selected for all other species within our study.

| **Test** |  | **Property** | **Value** | **Percentage** | **Source** |
| --- | --- | --- | --- | --- | --- |
| Human |  |  |  |  |  |
|  | Rose Bengal | Sensitivity | 18/23 | 78.6 | (98) |
|  |  | Specificity | 47/58 | 81.0% |  |
|  | PanBio Coxiella burnetii  immunoglobulin G [IgG] ELISA | Sensitivity | 95/134 | 70.9% | (99) |
|  |  | Specificity | 46/48 | 95.8% |  |
|  | ID Screen ® Rift Valley Fever  competition multi-species ELISA | Sensitivity | 40/40 | 100% | (100) |
|  |  | Specificity | 81/81 | 100% |  |
| Animal |  |  |  |  |  |
|  | Rose Bengal | Sensitivity | 87/96 | 90.6% | (101) |
|  |  | Specificity | 867/936 | 93.6% |  |
|  | ID Screen® Q Fever Indirect  Multi-species ELISA* | Sensitivity | 32/32 | 100% | (102) |
|  |  | Specificity | 178/178 | 100% |  |
|  | ID Screen ® Rift Valley Fever  competition multi-species ELISA* | Sensitivity | 40/40 | 100% | (103) |
|  |  | Specificity | 370/370 | 100% |  |

* No peer-reviewed comparative study for the evaluation of the ID Screen ELISA for Q fever in animals nor the ID Screen ® Rift Valley Fever competition multi-species ELISA compared to other diagnostic tests could be found, hence the diagnostic test producing company's internal validation values were used.
