# Supplementary material for: Seroprevalence and associated risk factors of brucellosis, Rift Valley fever and Q fever among settled and mobile agro-pastoralist communities and their livestock in Chad
Source: PLoS Negl Trop Dis. 2023 Jun 23;17(6):e0011395. doi: 10.1371/journal.pntd.0011395 (PMC10351688; doi:10.1371/journal.pntd.0011395)
Supplement: S1 R Script — (PDF) [file pntd.0011395.s013.pdf]

```
#Supporting information as an R statistical software script 1 for the
#statistical analysis of the manuscript entitled "Seroprevalence and associated
#risk factors of brucellosis, Rift Valley fever and Q fever among settled and
#mobile agro-pastoralists communities and their livestock in Chad"
#-.-.-.-.-.-.-.-.-.-.-.-.-.-.-.-.-.-.-.-.-.-.-.-.-.-.-.-.-.-.-.-.-.-.-.-.-.-.-.-.-.-.

library(rstan)

model <-"

data{
	int s;
	int n[s];
	int y[s];
	int params_SE[2];
	int params_SP[2];
}
parameters {
	real p[s];
	real Se;
	real Sp;
}
transformed parameters{
	real ap[s];
	for(i in 1:s)
		ap[i]=p[i]*Se+(1-p[i])*(1-Sp);
}
model{


	//Uniform (non-informative) prior for prevalence (p)
	p ~ beta(1,1);
	Se ~ beta(1,1);
	Sp ~ beta(1,1);

	params_SE[2] ~ binomial(params_SE[1], Se);
	params_SP[2] ~ binomial(params_SP[1], Sp);
	y ~ binomial(n, ap);

}
generated quantities{
	int Y_true[s];
	Y_true = binomial_rng(n, p);

}




"



### run model stan ###
stan_d<-list(y=c(43,20), #43/80 apparent positives, 20/100
```

```
            n=c(80,100),
            s=2,
            params_SE=c(100,95),
            params_SP=c(100,85))

initf2 <- function() { #initial values; now fixed, best to sample from assumed distribution.
  list(Sp=0.8,Se=0.9,p=rep(0.1,stan_d$s))
}

test2 = stan(model_code=model,
            data = stan_d, init=initf2,
            chains = 4, iter = 2000)

library(tidyverse)



summary(test2,pars=c("Y_true"))[1] %>%
  as.data.frame(.) %>%
  tbl_df() %>%
  add_rownames()


# by hand rogan gladen:

p = (ap + (sp-1))/(sp + (se-1))

ap=43/80
sp=85/100
se=95/100
(ap + (sp-1))/(sp + (se-1))

#vs:
39.2/80

summary(test2,pars=c("p"))[1] %>%
  as.data.frame(.) %>%
  tbl_df() %>%
  add_rownames()

summary(test2,pars=c("Se"))[1] %>%
  as.data.frame(.) %>%
  tbl_df() %>%
  add_rownames()

summary(test2,pars=c("Sp"))[1] %>%
  as.data.frame(.) %>%
  tbl_df() %>%
  add_rownames()
```
