# Supplementary material for: Seroprevalence and associated risk factors of brucellosis, Rift Valley fever and Q fever among settled and mobile agro-pastoralist communities and their livestock in Chad
Source: PLoS Negl Trop Dis. 2023 Jun 23;17(6):e0011395. doi: 10.1371/journal.pntd.0011395 (PMC10351688; doi:10.1371/journal.pntd.0011395)
Supplement: S2 R Script — (PDF) [file pntd.0011395.s014.pdf]

```
#Supporting information as an R statistical software script 2 for the
#statistical analysis of the manuscript entitled "Seroprevalence and associated
#risk factors of brucellosis, Rift Valley fever and Q fever among settled and
#mobile agro-pastoralists communities and their livestock in Chad"
#-.-.-.-.-.-.-.-.-.-.-.-.-.-.-.-.-.-.-.-.-.-.-.-.-.-.-.-.-.-.-.-.-.-.-.-.-.-.-.

# posterior: Beta(y + alpha, n-y + beta) for beta(alpha, beta) prior: alpha=1 beta=1
# https://en.wikipedia.org/wiki/Conjugate_prior

# example:
n=100
y=95

plot(density(rbeta(100000,n+1,n-y+1)))
abline(v=95/100)

summary(rbeta(1000000,n+1,n-y+1))




model1 <-"

data{
	int s;
	int n[s];
	int y[s];
	real params_SE[2];
	real params_SP[2];
}
parameters {
	real p[s];
	real Se;
	real Sp;
}
transformed parameters{
	real ap[s];
	real Se_t;
	real Sp_t;

	Se_t=Se;
	Sp_t=Sp;

	for(i in 1:s)
		ap[i]=p[i]*Se_t+(1-p[i])*(1-Sp_t);
}
model{
	y ~ binomial(n, ap);

	//Uniform (non-informative) prior for apparent prevalence (p)
	p ~ beta(1,1); //

	// see also: http://patricklam.org/teaching/conjugacy_print.pdf
```

```
        Se ~ beta(params_SE[1]+1, params_SE[1]-params_SE[2]+1);
        Sp ~ beta(params_SP[1]+1, params_SP[1]-params_SP[2]+1);

}
generated quantities{
        real Y_true[s];
        Y_true = binomial_rng(n, p);
}




"

library(rstan)
### run model stan ###
stan_d<-list(y=c(43,20), #43/80 apparent positives, 20/100
             n=c(80,100),
             s=2,
             #sesp=1,
             params_SE=c(100,95), #
             params_SP=c(100,85)) #9

initf2 <- function() { #initial values; now fixed, best to sample from assumed distribution.
  list(Sp=0.8,Se=0.9,p=rep(0.1,stan_d$s))
}

test1 = stan(model_code=model1,
             data = stan_d, init=initf2,
             chains = 4, iter = 2000)


summary(test1,pars=c("Y_true"))[1] %>%
  as.data.frame(.) %>%
  tbl_df() %>%
  add_rownames()
```
