# Supplementary material for: Seroprevalence and associated risk factors of brucellosis, Rift Valley fever and Q fever among settled and mobile agro-pastoralist communities and their livestock in Chad
Source: PLoS Negl Trop Dis. 2023 Jun 23;17(6):e0011395. doi: 10.1371/journal.pntd.0011395 (PMC10351688; doi:10.1371/journal.pntd.0011395)
Supplement: Attachment — Submitted filename: Seroprevalence and associated risk factors of brucellosis.docx [file pntd.0011395.s016.docx]

Seroprevalence and associated risk factors of brucellosis, Rift Valley fever and Q fever

among settled and mobile agro-pastoralist communities and their livestock in Chad

Abstract: check the journal word limitation (250-300 words) and adjust the abstract

Line 21: zoonosis

Line 31-33: remove the brackets and its contain

Line 34-36: credibility interval are given as [%CI:1-3]

Format the references as per the journal requirements

Line 79: maybe a mistake here. “reduce infertility”. I think you mean “reduce fertility”

Line 83: Vector-borne zoonotic viral disease

Figure 1: title: study sites. Are the pictures from www.d-maps open accessed and in concordance with the journal policy on publishable pictures?

Line 177: check the journal requirement for equations (eq.1)

Line 182-184: why exclude animals younger that two? Why exclude people younger than 14yrs?

Line 195: remove all trademarks from the manuscript

Line 199: add the name of the manufacturer of the Q fever ELISA test

Line 225: age value. Why is it <3 and ≤3 yrs? Since animal included were above two years, this value should be <3 and ≥3

Line 231: remove extra brackets after ggpair

Line 241-242: remove “(Supporting Fig 3 in SR1 shows the percentage of brucellosis, Q fever and RVF seropositive humans by age category and gender)” as it is not demographics

Line 258: figures should be cited in the main text, below the paragraph where the are used for the 1st time

Line 262-266: check the way seroprevalences are presented. 95%CI should be presented as 95%CI:[23-33%],

line 262-282: seroprevalence comparison by species and regions for each pathogen should come with statistical tests

line 307: remove the hyphen in Q fever throughout the text

line 339: something is missing after “within the same”

table 1: 95% CI is given as range
