# Supplementary material for: Seroprevalence and associated risk factors of brucellosis, Rift Valley fever and Q fever among settled and mobile agro-pastoralist communities and their livestock in Chad
Source: PLoS Negl Trop Dis. 2023 Jun 23;17(6):e0011395. doi: 10.1371/journal.pntd.0011395 (PMC10351688; doi:10.1371/journal.pntd.0011395)
Supplement: Attachment — Submitted filename: Abstract.docx [file pntd.0011395.s017.docx]

Abstract

Line 21, zoonosis not zoonoss

Line 22, Only a few studies, not Only few studies

Line 38, age for human Q fever must be replaced by the age for human Q fever

Line 46, to inform the public not to inform public

Introduction

Line 76, due to the lack not due the lack

Line 79 epididymitis not epidydimitis

Line 79 In animals, brucellosis not In animals brucellosis

Line 79 decline in milk not decline milk

Line 80, camels not camel

Line 82, orchiepididymitis not orchepididymitis

Line 83, vector-borne not vector-born

Line 84, was found not were found

Line 89, from livestock occurs not from livestock occur

Line 90, remove “through”

Line 104, “cause” should be replaced with “causes”

Line 110, remove “of”

Line 120, “life style” must be replaced with “lifestyle”

Line 121, “and often” must be replaced with “and is often”

Line 127, “are” should be replaced with “is”

Line 129, remove “in order”

Line 135, community-level not community level

Materials and methods

Line 147, province the bordering Central African Republic not province bordering Central African Republic

Line 150, “has” should be replaced with “have”

Line 159, Upon arrival at a study site not Upon arrival in a study site

Line 160, Line 162, the chief of the community not the chief of community

Line 163, “yet at some” should be replaced with “yet on some”

Line 166, back up not back-up

Line 167, “first level” should be replaced with “first-level”

Line 173, a simple random sampling not simple random sampling

Line 179, “lead” should be replaced with led

Line 179, add reference for ICC

Line 188, “the serum becoming” should be replaced with “the serum from becoming”

Line 200, remove “s” from “samples”

Line 201, “because of limited” should be replaced with “because of the limited”

Line 208, “is” should be replaced with “are”

Line 216, includes not include

Line 232, by a stepwise backward not by stepwise backwards

Line 234, as the selection criteria not as selection criteria

Line 235, remove “as” from as statistically significant

Results

Line 251 “From 966 human samples” should be replaced with “of

Line 252, “An overview on the number of humans and animals tested, number of diagnostically positive samples, apparent seroprevalence (AP) and the true seroprevalence (TP) per species and region for all three diseases is provided in Supporting Tab 3 in SR1” . should be replaced with “An overview of the number of humans and animals tested, number of diagnostically positive samples, apparent seroprevalence (AP) and the true seroprevalence (TP) per species and region for all three diseases were provided …..

Line 260, remove “a”

Line 276, and equine in Yao has the lowest

Line 292, “with the exception of sex” must be replaced with “except sex”

Line 293, “in the best fitted” should be replaced with “in the best-fitted”

Line 307, “was” should be replaced with “were”

Line 317, “with the exception of age” must be replaced with “except age”

Line 318, “in the best fitted” should be replaced with “in the best-fitted”

Line 327, insert “that” before small and remove “s” from factors

Line 338, “an increase of age of one year and a percent increase of RVF seropositivity of animals” should be replaced with “an increase in age of one year and a percent increase of RVF seropositivity of animals”

Line 346, remove comma “Sex,”

Discussion

Line 363, between the human and animal populations at the village level

Line 369, with a value of less than 1%

Line 377 add the name of kit of each pathogen and the cut of value

Line 378, with the Rose-Bengal test

Line 381, differs not differ

Line 389, during the abortion

Line 390, pastoralist not pastoralist’s

Line 390, goats not goat

Line 401, effect not affect

Line 408, Sokoto not Skoto

Line 412, a lower not lower

Line 431, constitute not constitutes

Line 439, correlation between not correlation of

Line 448, remove “s” from investments

Line 467, the difference

Line 469, particularly

Line 470, this was particularly the case of the recent RVF outbreak that occurred in 2016 on the Niger side of the Lake Chad region, which resulted

Line 473, humans have

Line 477, Animal-to-human direct transmission is associated

Line 481, … slaughtering, and transport of goods and people, as also happens in rural Chad.

Line 483, this finding or these findings

Line 519, with the aim of providing should be replaced with to provide

Line 520, to inform the public

Line 527, mobility of nomadic pastoralists
