# Supplementary material for: Seroprevalence and associated risk factors of brucellosis, Rift Valley fever and Q fever among settled and mobile agro-pastoralist communities and their livestock in Chad
Source: PLoS Negl Trop Dis. 2023 Jun 23;17(6):e0011395. doi: 10.1371/journal.pntd.0011395 (PMC10351688; doi:10.1371/journal.pntd.0011395)
Supplement: Attachment — Submitted filename: Responses to the reviewers.docx [file pntd.0011395.s018.docx]

**Responses to the reviewers (1/2)**

November 17^th^, 2022

Dear Reviewers,

We kindly thank each of you for your valuable time and expertise taken for the revision of our manuscript on the seroprevalence of three zoonotic diseases among Chadian agro-pastoralists and their livestock. We highly appreciate the value given to our study and the corresponding manuscript. We believe your comments and suggestions have generously bettered the scientific quality and readability of our manuscript, and hope our revised version of the manuscript will please the next readers better.

We have created two version of the revised manuscript, one in track change mode (named “Manuscript_Oezcelik_Revised_with_Highligted_Changes”) and the second with all revisions accepted (named “Manuscript_Oezcelik_Revised_clean”).

We have included all formatting, referencing and language edits suggested that derived from the two word documents attached to the review process (copy-pasted further down below) in the lines indicated, except if we had a reasoning not to implement the suggested change, where we then provide a short explanation.

Additionally, below, we address each comment made in the revision template of the journal (“Reviewer's Responses to Questions”) and mention the corresponding text passage that we amended in the track change version of the revised manuscript.

Many thanks once again and kind regards,

Ranya Özcelik

Representing the complete authors’ team

**From the revision part “Reviewer's Responses to Questions”**

**Authors’ response:** We provide a point-by-point response to all comments, formatted in blue.

1. **Methods**
   a. Reviewer #1: The authors do not include an explanation for how their cut-off of seropositive vs. seronegative samples for each disease. Please include this information
      i. We would like to note that the main objective of our study was to estimate the seroprevalence of the mentioned diseases using commercial diagnostic kits that have been authorized for use in the country. Therefore, we have used the cut-offs set by the manufactures without any changes for all different kits used to detect the targeted diseases. We have added this information at lines 214/215.
   b. Reviewer #3: the objectives were clearly articulated but the selection of the two districts must be justified. the population was not clearly described (the number of inhabitants and the total number of animals per site should be mentioned in the Materials and Methods section). the sampling process must be more detailed
      i. We added the requested information on lines 150 ff.
   c. Reviewer #x: Line 182-184: why exclude animals younger that two? Why exclude people younger than 14yrs?
      i. We have excluded animals younger than two years of age to maximize the chances of detecting antibodies of the targeted diseases, which are more likely to be detected in older animals than the very young ones. Furthermore, and based on our last experience in conducting similar studies, farmers usually hesitate to accept sampling younger animals as they think taking blood from them would make them weak, thus more exposed to diseases. People younger than 14 years are considered children, therefore, we have exclude them from the study such.
   d. Reviewer #x: Line 177: check the journal requirement for equations (eq.1)
      i. The equation format was checked according to the journal’s requirements.
   e. Reviewer #x: Line 199: add the name of the manufacturer of the Q fever ELISA test
      i. The information was added on lines 213ff.
2. Results
   a. Reviewer #1: The figures could be improved by: -Including the average value for the estimation on true seroprevalance in Fig 2 & Supporting Fig 2.
      i. The median estimate is already presented, we clarified this in the captions of the two figures (we guess it is Suppl. Fig 3, not 2)
   b. Reviewer #1: Providing a clear explanation of the units for each panel in Supporting Figs 1 & 2
      i. Amended.
   c. Reviewer #3: the results are clearly and completely presented. tables can be grouped into a single. figure 2 is unreadable
      i. We do not fully understand this comment. In the main manuscript, we only have one table. In the supporting results, we have 9 tables that we do not see how to group them into one single.
3. Conclusions
   a. Reviewer #1: - Starting at Line 396, the authors mention that the "higher odds of animal seropositive for brucellosis among females might be due to their prolonged life expectancy compared to males due to them being kep alive longer" however they do not explicitly state how their data supports this observation.
      i. This is correct, we added this caveat to the discussion (line 425).
   b. Reviewer #1: The authors do not discuss when the most recent outbreak that occured for each of these diseases. This could significantly contribute to the seroprevelance.
      i. Thank you for this valuable addition for the discussion. Discussion points for this were added to the discussion from lines 556 onwards.
   c. Reviewer #3: the conclusion were supported by the data presented. the limitations were not clearly described. the authors discuss how these data can be helpful to advance our understanding of the topic under study
      i. We thanks the reviewer for this positive comment.
4. Editorial and Data Presentation Modifications?
   a. Reviewer #1: Rift Valley fever virus was write with "Fever" capitalized, however it should be lower-case.
      i. Amended.
   b. There is no hyphan between agro-pastoralists. It should just be "agropastoralists"
      i. We have thus far observed both spelling versions in publications we have used to base our knowledge on and could not identify a distinguished difference in correct use of either version, hence propose to keep the spelling with the hyphen throughout our manuscript.
   c. Line 21: zoonoss should be "zoonoses"
      i. Amended.
   d. Line 22: Add "a" between only and few
      i. Amended.
   e. Line 23: Remove "three"
      i. Amended.
   f. Lines 48-55 would be best if it were truncated and left to the discussion/conclusions section
      i. Amended
   g. Line 72: Should read "aborted"
      i. Amended.
   h. Line 94: Please include relevant references explaining the high mortality rates in young animals
      i. We believe this information is correctly supported by the following reference already added in the manuscript, after the respective sentence; Gerdes GH. Rift Valley fever. Vol. 23, Rev. sci. tech. Off. int. Epiz. 2004.
   i. Line 98: should read "burnetti"
      i. Amended.
   j. Line 164-166: Can be removed since it isn't relevant to what was included in the study.
      i. Removed from the manuscript.
   k. 167: Remove word "being"
      i. Amended.
   l. Line 262: lower-case "human"
      i. Amended.
   m. Line 377: remove "of"
      i. Amended.
   n. Line 386: "Significantly"
      i. Amended.
   o. Line 448: remove "an"
      i. Amended.
   p. Line 467: "differences"
      i. Amended.
   q. Line 473: "humans"
      i. Amended.
   r. Line 478: "aborted"
      i. Amended.
   s. Line 481-482: Please clarify what you mean by "as is also happns in rural Chad"
      i. Text amended for clarification.
   t. Line 490-491 does not make sense
      i. Text removed from the manuscript.

**Reviewers comments/edits from the two attached word documents (2/2)**

**Authors’ response:** We modified all the editing suggestions and requirements from the reviewers with some exceptions justified below formatted in *blue and italic.*

**REVIEWER1**
Abstract

Line 21, zoonosis not zoonoss

Line 22, Only a few studies, not Only few studies

Line 38, age for human Q fever must be replaced by the age for human Q fever

- *We don’t agree to add the "the" due to consistency in the entire sentence.*

Line 46, to inform the public not to inform public

Introduction

Line 76, due to the lack not due the lack

Line 79 epididymitis not epidydimitis

Line 79 In animals, brucellosis not In animals brucellosis

Line 79 decline in milk not decline milk

Line 80, camels not camel

Line 82, orchiepididymitis not orchepididymitis

Line 83, vector-borne not vector-born

Line 84, was found not were found

Line 89, from livestock occurs not from livestock occur

Line 90, remove “through”

- *We replaced it with "by".*

Line 104, “cause” should be replaced with “causes”

- *We believe "can cause" should be kept.*

Line 110, remove “of”

- *We think that the "of" must stay.*

Line 120, “life style” must be replaced with “lifestyle”

Line 121, “and often” must be replaced with “and is often”

Line 127, “are” should be replaced with “is”

Line 129, remove “in order”

Line 135, community-level not community level

Materials and methods

Line 147, province the bordering Central African Republic not province bordering Central African Republic

- *We added a comma to clarify the meaning of the sentence*

Line 150, “has” should be replaced with “have”

Line 159, Upon arrival at a study site not Upon arrival in a study site

Line 160, Line 162, the chief of the community not the chief of community

Line 163, “yet at some” should be replaced with “yet on some”

Line 166, back up not back-up

Line 167, “first level” should be replaced with “first-level”

Line 173, a simple random sampling not simple random sampling

Line 179, “lead” should be replaced with led

Line 179, add reference for ICC

Line 188, “the serum becoming” should be replaced with “the serum from becoming”

Line 200, remove “s” from “samples”

Line 201, “because of limited” should be replaced with “because of the limited”

Line 208, “is” should be replaced with “are”

Line 216, includes not include

Line 232, by a stepwise backward not by stepwise backwards

Line 234, as the selection criteria not as selection criteria

Line 235, remove “as” from as statistically significant

Results

Line 251 “From 966 human samples” should be replaced with “of

Line 252, “An overview on the number of humans and animals tested, number of diagnostically positive samples, apparent seroprevalence (AP) and the true seroprevalence (TP) per species and region for all three diseases is provided in Supporting Tab 3 in SR1” . should be replaced with “An overview of the number of humans and animals tested, number of diagnostically positive samples, apparent seroprevalence (AP) and the true seroprevalence (TP) per species and region for all three diseases were provided …..

Line 260, remove “a”

Line 276, and equine in Yao has the lowest

Line 292, “with the exception of sex” must be replaced with “except sex”

Line 293, “in the best fitted” should be replaced with “in the best-fitted”

Line 307, “was” should be replaced with “were”

Line 317, “with the exception of age” must be replaced with “except age”

Line 318, “in the best fitted” should be replaced with “in the best-fitted”

Line 327, insert “that” before small and remove “s” from factors

Line 338, “an increase of age of one year and a percent increase of RVF seropositivity of animals” should be replaced with “an increase in age of one year and a percent increase of RVF seropositivity of animals”

Line 346, remove comma “Sex,”

Discussion

Line 363, between the human and animal populations at the village level

Line 369, with a value of less than 1%

Line 377 add the name of kit of each pathogen and the cut of value

Line 378, with the Rose-Bengal test

Line 381, differs not differ

Line 389, during the abortion

Line 390, pastoralist not pastoralist’s

Line 390, goats not goat

Line 401, effect not affect

Line 408, Sokoto not Skoto

Line 412, a lower not lower

Line 431, constitute not constitutes

Line 439, correlation between not correlation of

Line 448, remove “s” from investments

Line 467, the difference

Line 469, particularly

Line 470, this was particularly the case of the recent RVF outbreak that occurred in 2016 on the Niger side of the Lake Chad region, which resulted

Line 473, humans have

Line 477, Animal-to-human direct transmission is associated

Line 481, … slaughtering, and transport of goods and people, as also happens in rural Chad.

Line 483, this finding or these findings

Line 519, with the aim of providing should be replaced with to provide

Line 520, to inform the public

Line 527, mobility of nomadic pastoralists

**REVIEWER2**

Seroprevalence and associated risk factors of brucellosis, Rift Valley fever and Q fever

among settled and mobile agro-pastoralist communities and their livestock in Chad

Abstract: check the journal word limitation (250-300 words) and adjust the abstract

Line 21: zoonosis

Line 31-33: remove the brackets and its contain

Line 34-36: credibility interval are given as [%CI:1-3]

Format the references as per the journal requirements

Line 79: maybe a mistake here. “reduce infertility”. I think you mean “reduce fertility”

Line 83: Vector-borne zoonotic viral disease

Figure 1: title: study sites. Are the pictures from www.d-maps open accessed and in concordance with the journal policy on publishable pictures?

- *We have replaced the figure by a map made up by us.*

Line 177: check the journal requirement for equations (eq.1)

Line 182-184: why exclude animals younger that two? Why exclude people younger than 14yrs?

Line 195: remove all trademarks from the manuscript

Line 199: add the name of the manufacturer of the Q fever ELISA test

Line 225: age value. Why is it <3 and ≤3 yrs? Since animal included were above two years, this value should be <3 and ≥3

Line 231: remove extra brackets after ggpair

Line 241-242: remove “(Supporting Fig 3 in SR1 shows the percentage of brucellosis, Q fever and RVF seropositive humans by age category and gender)” as it is not demographics

Line 258: figures should be cited in the main text, below the paragraph where the are used for the 1st time

Line 262-266: check the way seroprevalences are presented. 95%CI should be presented as 95%CI:[23-33%],

line 262-282: seroprevalence comparison by species and regions for each pathogen should come with statistical tests

- *Response: We do not agree with this suggestion, because we have presented the 95% credibility intervals for all diseases and regions. Using these numbers, it is possible to directly evaluate the significance of the difference on a 5% level*

line 307: remove the hyphen in Q fever throughout the text

line 339: something is missing after “within the same”

table 1: 95% CI is given as range
