# Supplementary material for: Seroprevalence and associated risk factors of brucellosis, Rift Valley fever and Q fever among settled and mobile agro-pastoralist communities and their livestock in Chad
Source: PLoS Negl Trop Dis. 2023 Jun 23;17(6):e0011395. doi: 10.1371/journal.pntd.0011395 (PMC10351688; doi:10.1371/journal.pntd.0011395)
Supplement: Attachment — Submitted filename: REVIEW ARTICLE.docx [file pntd.0011395.s019.docx]

**Seroprevalence and associated risk factors of brucellosis, Rift Valley fever and Q fever among settled and mobile agro-pastoralist communities and their livestock in Chad**

I have reviewed the manuscript titled "Estimating the seroprevalence and risk factors for brucellosis, Rift Valley Fever and Q fever among agro-pastoralist communities and their livestock in Chad" by Ranya Özcelik et al. My general and specific comments are provided below. Please consider reformulating the manuscript based on these comments.

**General comments**

**Methodology and results**

- One potential limitation of the study is the selection of the two regions, which may limit the generalizability of the findings to other regions in Chad. Lack of justification for the selection of study regions and the author may need to provide a stronger justification for the selection of these two regions, such as explaining how they represent the population of Chad, or why they were chosen based on the availability of resources and infrastructure.
- Unclear explanation of sampling methodology: the author may need to provide more detail on how the first-level clusters were selected proportional to human population size, and how the second-level clusters were selected.
- Unclear exclusion criteria: The author may need to provide more justification for the exclusion criteria of pregnant women.
- Lack of detail on laboratory analyses: The author may need to provide more detail on the laboratory methods used.
- Using a single expected prevalence for all diseases, as was done in the methodology, assumes that the prevalence of all diseases is the same, which is not true according to the result. This can lead to an incorrect estimation of the required sample size and may result in underpowered or overpowered studies.
- It is important to use appropriate expected prevalence for each disease based on prior knowledge or evidence, or to estimate the expected prevalence from pilot studies or available data. This approach will help to ensure that the study is designed and conducted in a way that can accurately estimate the prevalence of each disease of interest. As the author mentioned in the introduction there are already previous study in chad and Using the prevalence of these studies as the expected prevalence can be a reasonable approach, particularly if the previous study was well-designed, conducted in a similar population, and is still considered current and relevant.
- The credibility intervals reported in the results are quite wide, which suggests that the sample sizes may not have been large enough to provide precise estimates of disease prevalence.
- In summary, the methodology and results reported in this study have several limitations and should be interpreted with caution. A more robust study design, based on prior knowledge and evidence, and with larger sample sizes, would be necessary to accurately estimate the prevalence of these zoonotic diseases.

**Specific comments**

- Line 16 : key words must be replaced with keywords
- Line 21, "endemic in numerous developing countries" can be replaced with "prevalent in many developing countries".
- Line 27, "true seroprevalence" can be replaced with "adjusted seroprevalence".
- Line 27: "was calculated applying" should be "were calculated applying"
- Line 28 : please insert “the” in “in study design”
- Line 28 : please insert “s” in “zoonotic disease”
- Line 30, "Q fever and RVF for both regions combined" can be changed to "Q fever and RVF combined for both regions".
- Line 31: "Q fever and RVF for both regions" should be "Q fever and RVF for both diseases"
- Line 33: "co-infection for animal brucellosis" should be "co-infection for human brucellosis"
- Line 39, "seems to present exceptionally" can be rephrased as "appears to have a low prevalence".
- Line 40: "between humans and animals living within" should be "between humans and animals living in"
- Line 41: "their health." should be "their health, highlighting the importance of a One Health approach."
- Line 51 : please insert “s” in goat and donkey
- Line 59: The sentence should read "Zoonotic diseases represent a major global threat to public health and the global economy. The World Health Organization (WHO) estimates that around one billion illnesses and millions of human deaths occur every year globally from zoonotic diseases (1). More than 60% of the emerging infectious diseases reported globally are of zoonotic origin, and around 75% of the latest 30 emerging human pathogens have originated in animal populations (2)."
- Line 67 : please insert “the” in “disrupted livelihoods of people”
- Line 68: The phrase "majorly on animal husbandry" should be corrected to "largely on animal husbandry."
- Line 79: "Delayed convalescence exist" should be corrected to "Delayed convalescence exists."
- Line 98: "varying endemic levels" should be "varying levels of endemicity"
- Line 102: "urine and faeces" should be "urine and feces"
- Line 105: "incidences" should be "cases"
- Line 105 : please remove : the from “Up to 30-52% of the severe Q fever”
- Line 106: "cause" should be "causes"
- Line 98 and 108: "Coxiella burnetti" should be "Coxiella burnetii" in the manuscript
- Line 110 : please remove “of” from “in many of sub-Saharan African countries”
- Line 117: "RVF" should be "Rift Valley fever".
- Line 126: There should be an apostrophe after "access" to indicate possession: "access'".
- Line 150 : insert the in “bordering Central African Republic”
- Line 154-155 : portion of the livestock population at the national
- Line 158-159 : housing opportunities for the research team
- Line 174 : clusters as second level
- Line 186: "We set m at 20 individuals to ensure a logistically optimized number of clusters to be sampled."
- Line 207 : STRANDARD should be corrected to STANDARD
- Line 207 : the Republic of Korea
- Line 257 : In bovine, sheep and goats more females
- Line 263 : An overview of the number of humans and animals tested, the number of diagnostically
- Line 305 : except sex
- Line 315 : Equine species (compared to cattle) and living in a village (compared to living in a camp) are negatively
- Line 330 : except age
- Line 352 : a percent increase in RVF seropositivity of
- Line 373 : presented as odds ratios
- Line 379 : including the association of diseases between the human and animal populations at the village level.
- Line 397 : This is confirmed by our results showing
- Line 404 : In pastoral settings,
- Line 429 : in all animal species together
- Line 440 : from the watercourse and well water
- Line 440-441 : with close contact with water
- Line 452: Data in our study suggest that the odds of human Q fever seropositivity are significantly lower with increasing age.
- Line 457-458: The risk factor analysis for Q fever seropositivity in animals revealed that small ruminants, compared to cattle, have significantly higher odds of being seropositive.
- Line 464 : considered as an investment
- Line 470: mobile pastoralists
